# A Graphene-Based Polymer-Dispersed Liquid Crystal Device Enabled through a Water-Induced Interface Cleaning Process

**DOI:** 10.3390/nano13162309

**Published:** 2023-08-11

**Authors:** Eun Mi Kim, Su Jin Kim, Go Bong Choi, Jaegeun Lee, Min Mo Koo, Jaewoong Kim, Young Won Kim, Jongho Lee, Jin Hyeok Kim, Tae Hoon Seo

**Affiliations:** 1 Green Energy and Nano Technology & R&D Group, Korea Institute of Industrial Technology (KITECH), Gwangju 61012, Republic of Korea; kimeunmi@kitech.re.kr (E.M.K.); ksjhy4@kitech.re.kr (S.J.K.); ywkim@kitech.re.kr (Y.W.K.); jholee@kitech.re.kr (J.L.); 2 School of Materials Science & Engineering, Chonnam National University, Gwangju 61186, Republic of Korea; 3 Korea Advanced Institute of Science and Technology (KAIST), Daejeon 34141, Republic of Korea; gbchoi@kaist.ac.kr; 4 School of Chemical Engineering, Pusan National University, Busan 46261, Republic of Korea; jglee@pusan.ac.kr; 5 Automotive Materials & Components R&D Group, Korea Institute of Industrial Technology (KITECH), Gwangju 61012, Republic of Korea; mmkoo@kitech.re.kr (M.M.K.); kjw0607@kitech.re.kr (J.K.); 6 Optoelectronic Convergence Research Center, Department of Materials Science and Engineering, Chonnam National University, Gwangju 61186, Republic of Korea

**Keywords:** adhesion, four-layer graphene, polymer-dispersed liquid crystal, smart window, water-induced interface cleaning

## Abstract

We report the use of four-layer graphene (4LG) as a highly reliable transparent conductive electrode (TCE) for polymer-dispersed liquid crystal (PDLC)-based smart window devices. The adhesion between 4LG and the substrate was successfully improved through a water-induced interface-cleaning (WIIC) process. We compared the performance of a device with a WIIC-processed 4LG electrode with that of devices with a conventional indium tin oxide (ITO) electrode and a 4LG electrode without a WIIC. With the application of the WIIC process, the PDLC smart window with a 4LG electrode exhibited reduced turn-on voltage and haze compared to 4LG without the WIIC process and characteristics comparable to those of the ITO electrode. The WIIC-processed 4LG electrode demonstrated enhanced electrical properties and better optical performance, leading to improved device efficiency and reliability. Furthermore, our study revealed that the WIIC process not only improved the adhesion between 4LG and the substrate but also enhanced the compatibility and interfacial interactions, resulting in the superior performance of the smart window device. These findings suggest that 4LG with WIIC holds great promise as a transparent conductive electrode for flexible smart windows, offering a cost-effective and efficient alternative to conventional ITO electrodes.

## 1. Introduction

The concept of a “net-zero-energy building”, which refers to the balance of emissions and energy in a building, attracts considerable attention and is considered a feasible solution instead of a distant future objective [1]. Several efforts have been made to reduce the energy consumed by building services such as ventilation, heating, and air conditioning [2,3]. Smart window technology is expected to play a crucial role in achieving eco-friendly energy savings in buildings because windows are among the most energy-efficient components. In particular, smart window devices based on PDLC offer various advantages, including a simple electrode-sandwiched design, straightforward fabrication process, quick response time, and durability [4,5,6]. Despite decades of steady and intensive research on liquid crystal (LC) coatings, polymer matrices, and ultraviolet aging, TCEs continue to pose major challenges. TCEs require low sheet resistance, high transparency, flexibility, and low cost. ITO has been commercially utilized as a TCE in PDLC-based smart windows, owing to its high optical transmittance and low sheet resistance. However, the ITO layer has several drawbacks such as rising prices due to the scarcity of indium, chemical instability towards acidic and basic sources, and high processing temperatures [7,8]. Furthermore, its application in flexible smart windows is severely hampered by its fragility when the substrate is bent or stretched by external force [9,10]. Therefore, alternative electrodes that can match or surpass ITO’s optical and electrical properties, without any disadvantages, are required. Numerous alternative materials such as carbon nanotubes [11], graphene [12,13,14,15], conductive polymers [16,17], and metal nanowires [18,19] have been explored and developed to replace ITO layers. Graphene has recently emerged as a promising alternative owing to its outstanding optical and electrical properties and thermal and chemical stabilities. Furthermore, graphene exhibits the flexibility required for use in smart windows. Pioneering studies have demonstrated PDLC-based smart window devices that employ graphene layers as TCEs on glass and other flexible plastic substrates. However, the lack of adhesion between graphene and its substrate can lead to local delamination, folding, and bending when exposed to a weak external force, resulting in the mechanical failure of graphene-based devices at their interface [20,21,22]. Despite the successful enhancement of adhesion to the substrate, graphene’s application as a TCE in smart windows has not yet surpassed the performance of conventional ITO-based smart windows. This is due to the fact that single-layer graphene (1LG) has a high sheet resistance of approximately 1.0 KΩ/sq [12,13,14]. Consequently, in order to commercialize a PDLC-based smart window with graphene, it is necessary not only to achieve strong adhesion properties between the substrate and the graphene interface, but also to apply an electrode, such as 4LG, with sheet resistance comparable to that of ITO. In this study, we report a graphene-based PDLC device using a 4LG TCE with a WIIC process. 4LG is the optimal layer for balancing the trade-offs between sheet resistance and transmittance, showing electrical characteristics comparable to ITO in contrast to 1LG. The WIIC process is simple and highly effective for eliminating impurities and improving the adhesion between graphene and the substrate. We demonstrated that the performance and stability of our graphene-based PDLC device are not only enhanced through the WIIC process but also comparable to those achieved using conventional ITO electrodes as conducting layers.

## 2. Materials and Methods

### 2.1. Synthesis of Graphene and Transfer to Glass Substrate

**Figure 1:**
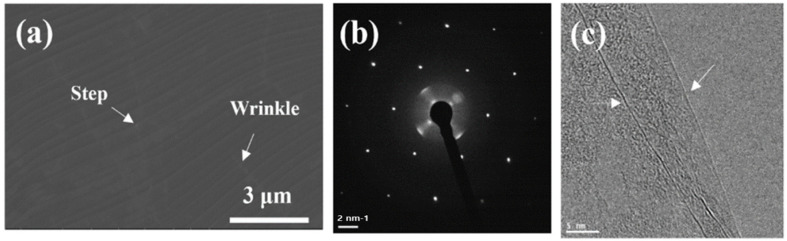
(**a**) SEM image, (**b**) selected area electron diffraction (SAED) pattern, and (**c**) high-resolution transmission electron microscopy (HRTEM) of graphene layer grown on Cu foils (White arrows indicating 1LG).

Low-pressure chemical vapor deposition (LPCVD) was utilized to grow a graphene layer on Cu foils (Nippon Mining, Tokyo, Japan) that were 35 µm thick. In order to reduce the surface roughness of the Cu foils and remove the impurity particles, for the first time, electro-chemical polishing (ECP) was conducted in a diluted 3H_3_PO_4_:H_2_O acid solution for 12 min at a constant voltage of 3.0 V using a Cu plate as the cathode and a Cu foil (200 mm × 200 mm) as the working electrode. Cu foils were then placed inside an 8-inch quartz tube and heated using a split-tube furnace. Meanwhile, the quartz tube was pumped down to 0.068 Torr, and 100 standard cubic centimeters per minute (sccm) of H_2_ gas permeated the reactor during the temperature increase to 1020 °C. Subsequently, the Cu foils were annealed for 60 min. CH_4_ of 10 sccm and H_2_ of 100 sccm were provided at 1020 °C for 18 min under a growth pressure of 0.13 Torr. Finally, the samples were swiftly cooled to room temperature with a cooling rate of 40 °C min^−1^ at an H_2_ flow rate of 100 sccm through removing the furnace from the substrate region. This is the optimal condition to achieve large and uniform graphene domains. Figure 1a shows the SEM image of the graphene layer synthesized on Cu foils. One can notice that the graphene layer is continuous, uniform, and clean without noticeable particles, whereas Cu surface steps, grain boundaries, and wrinkles are formed. Wrinkles associated with thermal coefficient difference between the Cu substrate and graphene formed during the cooling process across the Cu grain boundaries indicate that the graphene is continuous [23]. In addition, Figure 1b is a selected area electron diffraction (SAED) pattern displaying a typical hexagonal pattern for graphene. In Figure 1c, we also confirm that the graphene synthesized in this work is mostly composed of a single layer. Further details can be found in Ref. [24]. Following growth, the graphene was transferred to a glass substrate at 110 °C using a thermal release tape. We obtained 4LG using a four-step transfer procedure.

### 2.2. Water-Induced Interface Cleaning (WIIC) Process

**Figure 2:**
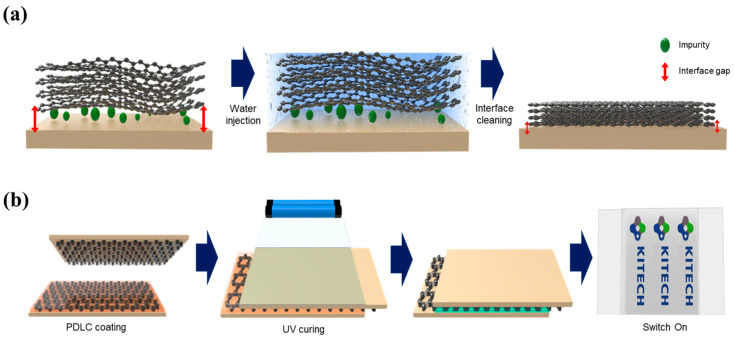
Schematic diagram of (**a**) WIIC process and (**b**) fabrication process of graphene-based PDLC device.

Figure 2a presents a schematic representation of the experimental design employed in this study. To enhance the adhesion between graphene and the glass substrate, we implemented the water-induced interface-cleaning (WIIC) process. The 4LG/glass configuration was immersed in deionized water for a duration of 1 min. Subsequently, the 4LG/glass sample underwent heat treatment in a chamber at 200 °C under vacuum conditions for 10 min, utilizing fast thermal processing equipment. The unique characteristics of graphene, specifically its smallest kinetic diameter [25,26,27,28], make it impermeable to gas molecules, preventing their passage through it. Consequently, water molecules confined between graphene and the substrate can only move laterally rather than vertically, allowing them to efficiently sweep away impurities. This leads to a remarkable improvement in the adhesion force, as the removal of impurities significantly enhances the bonding strength. Finally, in the last stage of the process, the sample was cooled to room temperature while maintaining an argon flow of 500 standard cubic centimeters per minute (sccm). Through these carefully orchestrated steps, the WIIC process was effectively applied, resulting in optimized adhesion properties between graphene and the glass substrate.

### 2.3. Fabrication of PDLC Device

To fabricate the PDLC devices, we prepared three different transparent conductive electrodes (TCEs): (1) conventional ITO, (2) four-layer graphene without the WIIC process (4LG w/o WIIC), and (3) four-layer graphene with the WIIC process (4LG w/WIIC). The PDLC material, consisting of a liquid crystal, pre-polymer, and photo-initiator, was obtained from Qingdao QY Liquid Crystal Co., Ltd., Qingdao, China (QYPDLC-100). To achieve a uniform PDLC device thickness, we incorporated 20 μm silica microspheres as spacers. The PDLC and microsphere spacer solution were carefully deposited onto the bottom electrode of the glass substrate. Next, another sample with the same electrode was placed on top to form a sandwich configuration with a defined gap. Finally, the PDLC mixture was cured through exposing the device to a UV lamp with an intensity of 1 mW/cm^2^ at 365 nm for a duration of 60 s. Figure 2b shows a schematic of the fabricated PDLC device with 4LG as the TCE.

### 2.4. Characterization

Raman spectroscopy (Renishaw, Hongkong, China) was used to determine the number of layers and evaluate the quality of the graphene. The 514 nm line of an Ar-ion laser served as the excitation source for the Raman measurements. Additionally, we utilized field-emission scanning electron microscopy (FESEM, Thermo Fisher Scientific, Waltham, MA, USA) to investigate the surface morphology of graphene transferred to a SiO_2_/Si substrate. To validate the uniformity and electrical properties, we conducted sheet resistance mapping measurements using a non-contact sheet resistance meter and a Hall measurement system. For optical characterization, we measured the transmittance and haze as functions of various driving voltages using a UV-Vis-NIR spectrophotometer (Lambda750, Perkin Elmer, Hongkong, China) and a haze meter (Nippon Denshoku, NDH5000, Tokyo, Japan), respectively. These comprehensive analyses allowed us to thoroughly examine the characteristics and performance of graphene in our smart window devices. High-resolution transmission electron microscopy (HRTEM, Thermo Fisher Scientific, Waltham, MA, USA) investigations were performed utilizing a FEI TITAN G2, equipped with an image Cs corrector and a monochromator. The instrument was operated at an optimized acceleration voltage of 60 kV to prevent knock-on damage to the graphene.

## 3. Results

### 3.1. Number of Graphene Layer

**Figure 3:**
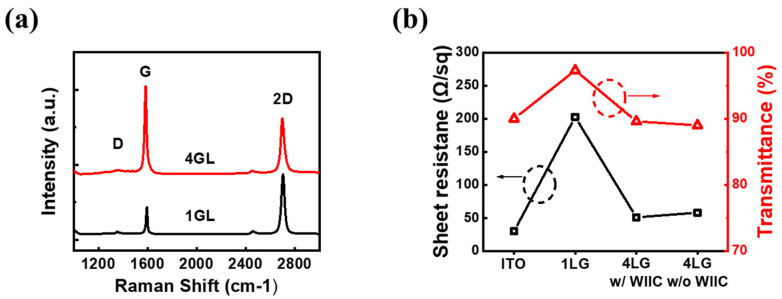
(**a**) Raman spectra of 1LG and 4LG transferred to a SiO_2_/Si substrate. (**b**) Transmittance and sheet resistance of ITO, 1LG, 4LG with WIIC, and 4LG without WIIC, respectively.

The two crucial considerations for the use of TCEs in PDLC devices are optical transmittance and sheet resistance, because these two parameters significantly impact the device’s performance. Achieving a balance between these parameters is of utmost importance when selecting transparent conductive electrodes (TCEs). While the use of 1LG is desirable due to its high optical transmittance, it comes with the drawback of high sheet resistance. As a potential solution, we sought to strike a balance between sheet resistance and transmittance through increasing the number of graphene layers. Drawing from our expertise, we hypothesized that four-layer graphene (4LG) could serve as an ideal TCE in PDLC devices, offering both high transmittance and low sheet resistance. To verify this hypothesis, our first step involved ensuring the successful transfer of high-quality 1LG and 4LG onto substrates. We meticulously transferred 1LG and 4LG onto SiO_2_/Si substrates and performed Raman spectroscopy to thoroughly investigate their quality and quantity, shedding light on their suitability as TCE candidates for PDLC devices. In Figure 3a, we present the Raman spectra obtained using the 514 nm line of an Ar-ion laser. The Raman spectrum of 1LG reveals two prominent peaks: the G band located at 1594 cm^−1^ and the 2D band at 2703 cm^−1^. These peaks demonstrate typical characteristics of monolayer graphene, including a remarkably sharp Lorentzian peak with a narrow full width at half maximum (FWHM) of 30 cm^−1^, a symmetrical shape, and a 2D-to-G intensity ratio exceeding two. Interestingly, as the number of graphene layers increases, the 2D peak broadens and diminishes in intensity, whereas the G peak becomes more pronounced [29,30]. The spectra of 4LG had a lower 2D-to-G intensity ratio (0.79) and a larger FWHM (38 cm^−1^) than those of 1LG. Moreover, the so-called defects-induced or disorder-induced D-band peak at 1350 cm^−1^ is minuscule, indicating that 4LG is high quality with few defects. The transmittances and sheet resistances of the four TCEs are shown in Figure 3b. The transmittances of the conventional ITO, 1LG, and 4LG without the WIIC process and 4LG with the WIIC process at 550 nm were 90.0%, 97.5%, 89.6%, and 89.8%, respectively. As expected, the 1LG TCE exhibits the highest transmittance. Nevertheless, the transmittance of the 4LG TCE is comparable to that of the ITO TCE. The sheet resistance was also determined. Sheet resistance measurements were performed using a Hall measurement system. The obtained sheet resistance values of conventional ITO, 1LG, 4LG without WIIC, and 4LG with WIIC are 30 ± 2 Ω/sq, 202 ± 10 Ω/sq, 49 ± 4 Ω/sq, and 57 ± 2 Ω/sq, respectively. The sheet resistance of 4LG with the WIIC process was slightly higher than that of 4LG without the WIIC process. This can be attributed to the fact that some defects in 4LG are linked to oxygen groups during the WIIC process. Overall, the 4LG TCEs simultaneously exhibited high transmittance and low sheet resistance, which is consistent with our hypothesis. Due to the fact that monolayer graphene has an opacity of 2.3% at the wavelength of 550 nm, the transmittance of 4LG without the WIIC process is about 89.6%, which deviates slightly from the predicted value of 90.6%. However, this is a typical trend since CVD-grown graphene contains a small amount of bilayer or trilayer, depending on growth conditions. When the WIIC process is used to apply 4LG on glass substrate, transmittance increase by approximately 0.2% compared with that of 4LG without WIIC process. This might be because impurities in the interface of the artificially constructed weak van der Waals stacked layers have been removed. Consequently, the transmittance of 4LG with the WIIC process is comparable to that of ITO.

### 3.2. Effect of WIIC

**Figure 4:**
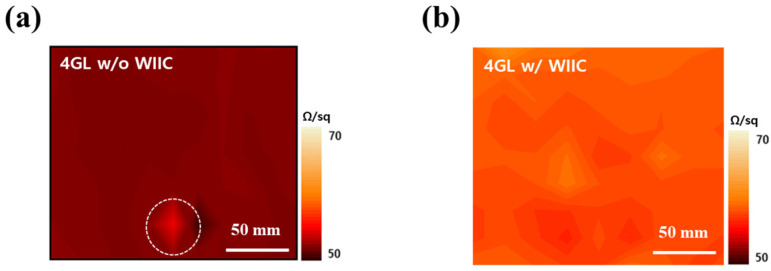
Four-point mapping images of (**a**) 4LG without WIIC process and (**b**) 4LG with WIIC process at a 200 mm × 200 mm area.

**Figure 5:**
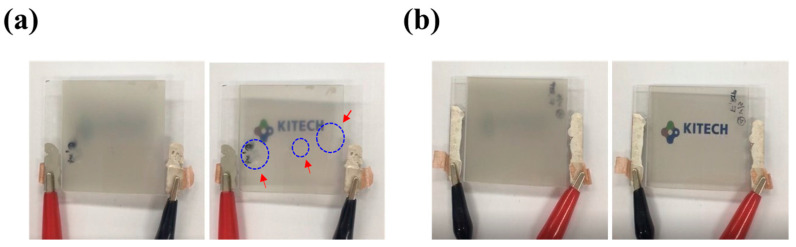
Optical images for PDLC devices of (**a**) 4LG without WIIC process and (**b**) 4LG with WIIC process in on/off state.

**Figure 6:**
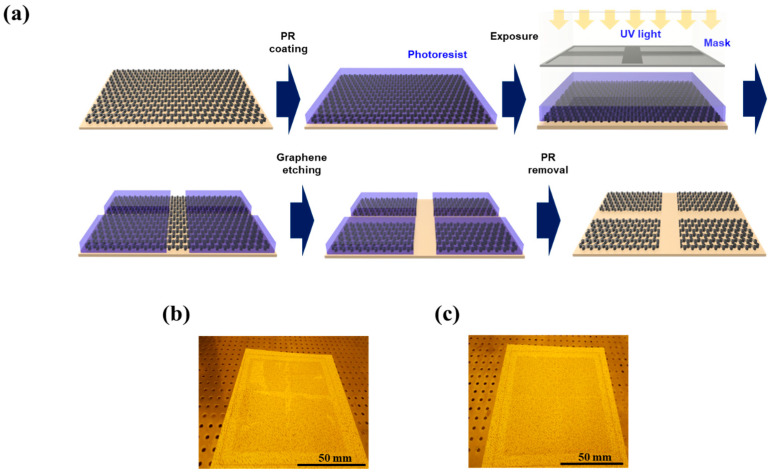
(**a**) Schematic diagram of fabrication process for the patterned 4LG using photolithography and etching. Optical images of the patterned 4LG (**b**) without WIIC process and (**c**) with WIIC process.

Our second strategy to improve the performance of graphene-based PDLC devices was to employ the WIIC process to remove impurities at the interface between the TCE and substrate. We firmly believe that the WIIC process offers two key advantages that greatly contribute to device improvement. Firstly, it ensures the remarkable uniformity of the TCE across the entire study area. Secondly, it significantly enhances the adhesion between the TCE and the substrate, promoting overall device performance. The uniformity of the TCE was already evident during the sheet resistance measurements, where the sheet resistance of 4LG without the WIIC process and 4LG with the WIIC process were recorded as 49 ± 4 Ω/sq and 57 ± 2 Ω/sq, respectively. Remarkably, the standard deviation of the sheet resistance of 4LG with the WIIC process was only half of that observed for 4LG without the WIIC process. To further corroborate the uniformity of 4LG on a larger scale, we meticulously performed four-point mapping over a substantial 200 mm × 200 mm area for both 4LG with and without the WIIC process, as depicted in Figure 4a,b. The resulting sheet resistance value for 4LG with the WIIC process was measured as 58 ± 2 Ω/sq, slightly higher than the value of 51 ± 5 Ω/sq obtained for 4LG without the WIIC process. This result aligned seamlessly with the Hall measurements, providing further evidence of the uniformity achieved through the WIIC process. Despite the slight increase in sheet resistance brought about by the WIIC procedure, it is important to emphasize that the entire area exhibited a consistent and uniform sheet resistance value. This can be attributed to the effective elimination of impurities, as indicated by the white circle in Figure 4a, and is likely a significant factor responsible for the observed improvements in the device performance. The effect of the WIIC process is further supported by the on/off optical images shown in Figure 5a,b. The optical image of the PLDC device with a 4LG electrode without the WIIC process at an applied voltage is unclear and shows the graphene-burning phenomenon resulting from the high-power consumption of graphene [31,32], as marked by the blue circle in Figure 5a. Because of the current crowding induced by impurities and adhesion between the graphene and the substrate at 60 V injection, an excessive quantity of power builds up under the graphene electrode in the device, destroying the graphene with Joule heat. As illustrated in Figure 5b, in the case of the 4LG electrode following the application of the WIIC process, the device in the ON state was nearly transparent and uniform across the entire operation region. This clearly indicates that the 4LG electrode with the WIIC process as a transparent conductive layer can act as an efficient lateral current diffusion channel. The WIIC process effect can be evaluated using the above results obtained from the PDLC device, but the adhesion between the 4LG electrode and the substrate could not be verified. In order to ascertain the effectiveness of the WIIC process in enhancing the adherence of the 4LG electrode to the substrate, we undertook the fabrication of patterned graphene using photolithography and etching, spanning a considerable area of 200 mm × 200 mm, as depicted in Figure 6a. The process involved transferring the chemical vapor deposition (CVD)-grown graphene films four times onto a glass substrate to form the patterned graphene. Subsequently, the patterned region was safeguarded through applying a protective photoresist (PR) as an etchant mask, and the graphene was selectively patterned using an inductively coupled plasma reactive-ion etcher with O_2_ plasma. Lastly, the PR was meticulously removed using acetone. Optical images of the resulting patterned graphene without and with the WIIC process are shown in Figure 6b,c, respectively. The surface of patterned graphene without a WIIC process is readily and partially peeled-off during fabrication owing to the lack of chemical reactivity of graphene, which consists of the C-C bond of sp^2^ hexagonally arranged carbon atoms with no dangling bonds [20,21,22,33]. When the WIIC process was applied, we observed a cleanly patterned graphene surface without peeling. This is as a result of the fact that the adhesion of graphene to the substrate was stronger than its attachment to the PR, which is indicative of enhanced adhesion. We believe that this observed effect can be ascribed not only to the successful removal of impurities, but also to the interaction of an oxygen group with the graphene defect, resulting in a transformation of its inherent hydrophobicity into hydrophilicity [34].

### 3.3. Performance of PDLC Device

**Figure 7:**
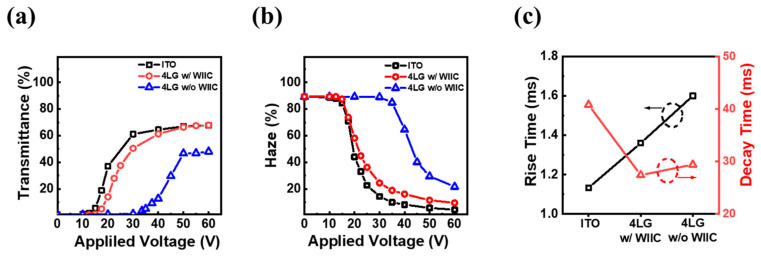
(**a**) Transmittance (at 550 nm) and (**b**) haze according to applied voltage in PDLC devices of ITO, 4LG with WIIC process, and 4LG without WIIC process, respectively. (**c**) Rise and decay time for PDLC devices of three different TCEs investigated in this work.

The properties of the PDLC device were examined to understand the potential of the WIIC process. Figure 6a,b show the transmittance at 550 nm and haze of the PDLC devices with conventional ITO, 4LG without the WIIC process, and 4LG with the WIIC process, respectively, as a function of the applied voltage. All devices scattered or reflected almost all the incident light when turned off. As the applied voltage increased, more incident light was transmitted through the device, reaching a saturation state at a critical voltage. As shown in Figure 7a, the transmittance of the device with the conventional ITO electrode rapidly increased at injection voltages exceeding 15 V and reached a maximum transmittance of 68% at 60 V. For the bare 4LG electrode, the transmittance increased at driving voltages above 35 V and reached a maximum transmittance of 48% at 60 V. The relatively high turn-on voltage and low transmittance associated with the bare graphene electrode were due to insufficient current spreading due to poor contact between graphene and the substrate. When the WIIC process was applied to the bare 4LG electrode, the transmittance-voltage curves revealed a considerable reduction in the turn-on voltage of 17 V and an increase in the maximum transmittance of 20% compared to those of the 4LG electrode-based device without the WIIC process. This is attributed to the improvement in the adhesion level between the graphene layer and substrate, which indicates that the 4LG electrode with the WIIC process serves as the current-spreading electrode of the PDLC device. The haze as a function of the injection voltage for the PDLC devices with the three different TCEs, as shown in Figure 7a, is illustrated in Figure 7b. The haze values of ITO, 4LG without WIIC, and 4LG with WIIC at 60 V were measured to be 5, 22, and 10%, respectively. Through applying the WIIC process, the haze was significantly reduced by approximately 12% compared with that of the 4LG electrode without the WIIC process. As shown in Figure 7a,b, even if the sheet resistance increased after utilizing the WIIC process, this was due to the improved interfacial adhesion. However, the haze and turn-on voltages for the PDLC device with the 4LG electrode and the WIIC process were still slightly higher than those of the PDLC device with the conventional ITO electrode owing to the relatively high sheet resistance. If the opto-electrical performance of graphene is further improved through optimizing the graphene transfer and improving the electrical properties of the graphene layer in the CVD synthesis, it will be suitable for use as a TCE in flexible devices owing to the lack of flexibility in ITO. Figure 7c illustrates the temporal characteristics of all the PDLC devices. During the off-to-on and on-to-off voltage transitions, the rise and decay times were defined as the times required for the transmittance to increase from 10% to 90%, respectively. Rise times of ITO, 4LG without WIIC, and 4LG with WIIC were 1.1, 1.4, and 1.6 ms, respectively, and their decay times 41, 27, and 29 ms, respectively. The rise time of 4LG with WIIC was shorter than that of 4LG without WIIC owing to the reduced sheet resistance. While all other material properties are constant, it is widely known that the decay time increases with the droplet size. We believe that the decrease in the decay time of PDLC devices with graphene electrodes is a result of smaller LC droplets in the polymer matrix [14]. Details of the decay time results will be discussed in future studies.

## 4. Conclusions

We investigated the effect of adhesion on the performance of 4LG as a TCE in PDLC devices. The successful implementation of the WIIC process effectively increased the adhesion between 4LG and the substrate. The sheet resistance of 4LG with the WIIC process used in this work was measured to be 58 ± 2 Ω/sq, exhibiting a remarkably uniform sheet resistance across the entire area. To further confirm the positive impact of the WIIC process, we fabricated a PDLC device with the 4LG electrode incorporating the WIIC treatment. The device created with the WIIC process exhibited significant improvements in various performance metrics. Notably, it demonstrated a considerable 17 V reduction in turn-on voltage, a remarkable 20% enhancement in maximum transmittance, and an appreciable 12% reduction in haze compared to the device created without the WIIC process. Our findings underscore the potential of graphene with increased adhesion and ultra-low resistance as a TCE in flexible devices, owing to its enhanced stability and superior performance characteristics. The successful integration of the WIIC process opens up exciting opportunities for leveraging 4LG as a highly reliable and efficient TCE in a wide range of flexible electronic and optoelectronic applications.

## Data Availability

The data presented in this study are available on request from the corresponding author.
